# Systemic Inflammation and Nutritional Index as a prognostic marker in endometrial cancer

**DOI:** 10.17305/bb.2026.14354

**Published:** 2026-06-10

**Authors:** Nagihan Kolkıran, Atike Pınar Erdoğan, Mustafa Şahbazlar, Mustafa Şahin, Ferhat Ekinci

**Affiliations:** 1 Department of Medical Oncology, Faculty of Medicine, Manisa Celal Bayar University, Manisa, Türkiye; 2 Department of Internal Medicine, Afyonkarahisar State Hospital, Afyonkarahisar, Türkiye

**Keywords:** Endometrial cancer, prognostic biomarker, survival outcomes, systemic inflammation, nutritional status, risk stratification

## Abstract

The clinical course of endometrial cancer exhibits substantial variability, underscoring the need for additional biomarkers to enhance risk stratification accuracy. This study aimed to evaluate the prognostic utility of a novel composite index, integrating systemic inflammatory and nutritional parameters, herein termed the Systemic Inflammation and Nutritional Index (SINI), in patients diagnosed with endometrial cancer. This retrospective study included 104 patients with histologically confirmed endometrial cancer. Clinical, pathological, and laboratory data were extracted from institutional medical records. SINI was computed using the formula: platelet count × neutrophil count / lymphocyte count × serum albumin. Receiver operating characteristic (ROC) curve analysis was employed to ascertain the optimal cut-off value, thereby stratifying patients into low and high SINI cohorts. Survival outcomes were evaluated using Kaplan–Meier analysis, and prognostic factors were identified via Cox regression analysis. In multivariable Cox regression analysis, treating SINI as a continuous variable, SINI remained significantly associated with overall survival (HR: 1.656; 95% CI: 1.203–2.279; *P ═* 0.002). Exploratory ROC analysis identified a potential cutoff value of 159.3 for patient stratification. In subsequent cutoff-based analyses, patients with SINI >159.3 demonstrated significantly worse overall survival in multivariable analysis (HR: 2.89; 95% CI: 1.30–6.43; *P ═* 0.009). Kaplan–Meier analysis further corroborated these findings, revealing significantly diminished overall survival in the high SINI group compared with the low SINI group (log-rank *P ═* 0.006). The SINI was associated with survival outcomes in endometrial cancer. As a novel composite biomarker derived from routinely available and cost-effective clinical parameters, SINI offers a potentially practical and accessible tool for refined risk stratification in endometrial cancer patients.

## Introduction

Endometrial carcinoma (EC) stands as the most prevalent gynecologic malignancy in high-income nations, exhibiting a consistently rising incidence attributed to aging demographics and escalating obesity rates [1, 2]. While outcomes for early-stage disease are generally favorable, a substantial proportion of patients experience recurrence and ultimately poor survival [2, 3]. Traditional clinicopathological factors, such as International Federation of Gynecology and Obstetrics (FIGO) stage, histological subtype, tumor grade, and lymphovascular space invasion (LVSI), form the bedrock of risk stratification. However, these parameters inadequately capture the marked heterogeneity in clinical outcomes observed among EC patients [2]. Consequently, there has been increasing investigation into circulating peripheral blood biomarkers that reflect disease burden in EC. Cancer antigen 125 (CA125), for instance, has demonstrated correlation with extra-uterine disease, lymph node metastasis, and advanced stage, with elevated levels linked to poorer survival outcomes in gynecological cancers [4]. Nevertheless, these individual markers only partially elucidate the intricate biological complexity of the disease.

The pivotal role of systemic inflammation in shaping tumor characteristics and outcomes has garnered increasing recognition. Tumor-associated inflammation can originate from obesity-related cytokine production and chronic low-grade inflammatory signaling, thereby fostering cellular proliferation, reducing apoptosis, and ultimately facilitating tumor development and progression [5]. Furthermore, systemic inflammation has been associated with adverse clinical outcomes, reflecting alterations in circulating immune and hematologic parameters in EC patients [5, 6]. Peripheral blood components, particularly neutrophils, lymphocytes, and platelets, have thus emerged as accessible biomarkers that mirror the dynamic interplay between tumor biology and host immune response [6]. These components serve as accessible indicators of the host inflammatory response. Neutrophils are known to promote tumor progression through the secretion of pro-inflammatory mediators, whereas lymphocytes are central to anti-tumor immunity. Therefore, an imbalance between these components signifies a shift towards a tumor-promoting inflammatory state [7, 8]. Building upon these biological mechanisms, several inflammation-based biomarkers derived from routine blood tests have been investigated in EC. Among these, composite indices such as the systemic immune-inflammation index (SII), neutrophil-to-lymphocyte ratio (NLR), and monocyte-to-lymphocyte ratio have been explored for their potential to reflect systemic inflammatory status and tumor aggressiveness [8, 9].

**Table 1:** Demographic and clinicopathologic characteristics of patients

		**Total *n* (%)**	**SINI ≤159.3 *n* (%)**	**SINI >159.3 *n* (%)**	***P*-value**
**Age**	Mean± SD (min–max)	61.6±10.6 (30–84)	57.5±10.3 (35–78)	62.9±10.4 (30–84)	0.022
ECOG					0.678	
	0–1	90 (86.5)	24 (88.9)	66 (85.7)		
	2–3	14 (13.5)	3 (11.1)	11 (14.3)		
Operation					0.348	
	Yes	90 (86.5)	25 (92.6)	65 (84.4)		
	No	14 (13.5)	2 (7.4)	12 (15.6)		
Adjuvant Chemotherapy					0.823	
	Yes	52 (50.0)	13 (48.1)	39 (50.6)		
	No	52 (50.0)	14 (51.9)	38 (49.4)		
Adjuvant Radiotherapy					0.758	
	Yes	59 (56.7)	16 (59.3)	43 (55.8)		
	No	45 (43.3)	11 (40.7)	34 (44.2)		
Lymph node dissection					0.218	
	Yes	75 (72.1)	17 (63.0)	58 (75.3)		
	No	29 (27.9)	10 (37.0)	19 (24.7)		
Stage at Diagnosis					0.024	
	1	18 (17.3)	7 (25.9)	11 (14.3)		
	2	13 (12.5)	7 (25.9)	6 (7.8)		
	3	39 (37.5)	8 (29.6)	31 (40.3)		
	4	34 (32.7)	5 (18.5)	29 (37.7)		
Histology					0.025	
	Endometrioid	62 (59.6)	21 (77.8)	41 (53.2)		
	Non-endometrioid	42 (40.4)	6 (22.2)	36 (46.8)		
Grade					0.039	
	1	17 (16.3)	8 (29.6)	9 (11.7)		
	2	47 (45.2)	13 (48.1)	34 (44.2)		
	3	40 (38.5)	6 (22.2)	34 (44.2)		
LVSI					0.094	
	Yes	36 (34.6)	10 (37.0)	26 (33.8)		
	No	25 (24.0)	10 (37.0)	15 (19.5)		
	Unknown	43 (41.3)	7 (25.9)	36 (46.8)		
Myometrial invasion					0.100	
	≤ 50%	30 (28.8)	12 (44.4)	18 (23.4)		
	> 50%	66 (63.5)	14 (51.9)	52 (67.5)		
	Unknown	8 (7.7)	1 (3.7)	7 (9.1)		
*De novo* metastasis	26 (25.2)	3 (11.5)	23 (29.9)	0.063		
Liver metastasis	17 (16.3)	3 (11.5)	14 (18.2)	0.551		
Lung metastasis	23 (22.1)	5 (18.5)	18 (23.4)	0.601		
Bone metastasis	8 (7.7)	0 (0.0)	8 (10.4)	1.000		
Brain metastasis	2 (1.9)	0 (0.0)	2 (2.6)	1.000		
BMI (kg/m^2^)	Mean± SD	31.9±6.6 (17.6–47.6)	31.4 ± 5.9 (23.7–44.7)	32.0 ± 6.9 (17.6–47.6)	0.601	

Data are presented as mean ± standard deviation or counts (percentages). Comparisons were performed using the independent samples *t*-test or chi-square test, as appropriate. **Abbreviations:** BMI, body mass index; ECOG, Eastern Cooperative Oncology Group performance status; LVSI, lymphovascular space invasion; SD, standard deviation; SINI, Systemic Inflammation and Nutritional Index.

More recently, composite indices that integrate inflammatory, immune, and nutritional parameters have attracted considerable attention. The hemoglobin–albumin–lymphocyte–platelet (HALP) score, for example, has been proposed as a comprehensive biomarker reflecting tumor–host interaction and has demonstrated independent prognostic value for recurrence and survival outcomes in EC [10]. These findings suggest that combining multiple systemic parameters may provide a more accurate assessment of disease biology than single biomarkers alone [10].

Despite these advancements, currently available biomarkers still capture only limited facets of the complex interplay between tumor biology and systemic host conditions. In this context, we developed a novel composite biomarker, the systemic inflammation and nutritional index (SINI), calculated from platelet count, neutrophil-to-lymphocyte ratio, and serum albumin levels. By integrating markers of tumor-promoting inflammation (platelets and neutrophils), host immune response (lymphocytes), and nutritional status (albumin), SINI was designed to encapsulate multiple biological dimensions relevant to cancer progression. This study aimed to evaluate the prognostic significance of SINI for survival outcomes and its utility in risk stratification for endometrial cancer.

## Materials and methods

This study was reported in accordance with the Strengthening the Reporting of Observational Studies in Epidemiology (STROBE) guidelines.

### Patient and data collection

Patients with histologically confirmed endometrial cancer treated at Manisa Celal Bayar University between March 2011 and October 2024 were retrospectively analyzed. Inclusion criteria comprised patients aged ≥18 years with available survival data, laboratory parameters obtained at diagnosis for SINI calculation, and complete baseline clinicopathological data. Patients with missing laboratory data or follow-up outside our institution were excluded. Due to the retrospective nature of the study, certain clinicopathological variables, such as LVSI and myometrial invasion, were not consistently available. These were retained as “unknown” categories in descriptive and univariate analyses but were not included in multivariable models. Data retrieved from medical records included age, Eastern Cooperative Oncology Group (ECOG) performance status, presenting symptoms, body mass index, stage, histology, grade, Ki-67 index, LVSI, myometrial invasion, nodal and metastatic status, treatment modalities, and baseline laboratory parameters (neutrophils, lymphocytes, albumin, platelets, carcinoembryonic antigen, cancer antigen 125).

### SINI measurement

SINI was calculated using the formula: SINI ═ (platelet count × neutrophil count) / (lymphocyte count × serum albumin level). The component (platelet × neutrophil / lymphocyte) corresponds to the SII as reported in the literature [11]. Platelet, neutrophil, and lymphocyte counts were recorded as ×10^9^/L, and albumin as g/dL. Laboratory parameters used for SINI calculation were obtained at the time of diagnosis, prior to the initiation of any cancer-directed treatment, including surgery, chemotherapy, or radiotherapy. To enhance interpretability, SINI values were divided by 100 before statistical analyses; thus, hazard ratios reflect the risk associated with each 100-unit increase in SINI.

### Outcomes

The primary endpoint was overall survival (OS), defined as the time from diagnosis to death or last follow-up. The secondary endpoint was progression-free survival (PFS), defined as the time from diagnosis to disease progression, recurrence, or death from any cause.

### Ethical statement

The study protocol was approved by the Health Sciences Ethics Committee of Manisa Celal Bayar University Faculty of Medicine (approval no. 20.478.486/1219; March 9, 2022). All procedures were performed in accordance with the ethical standards of the Declaration of Helsinki.

**Figure 1.**
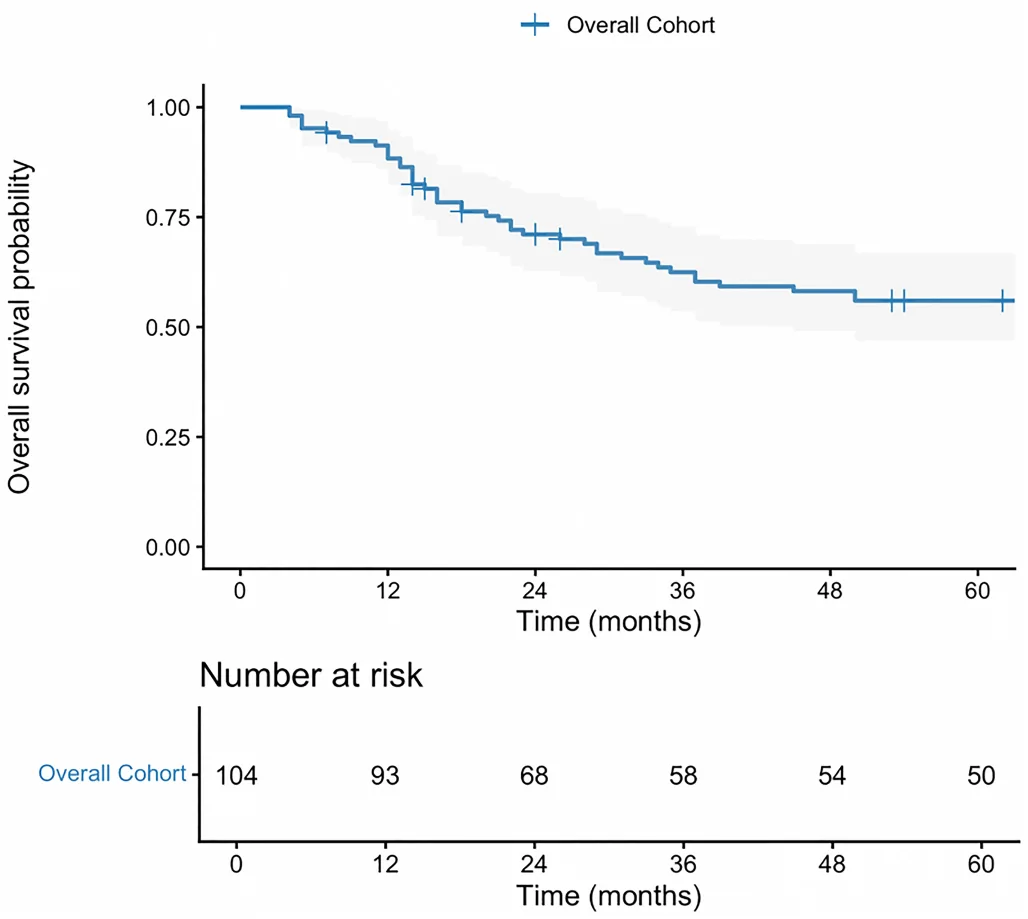
**OS in the entire endometrial cancer cohort.** Kaplan–Meier estimate of OS among 104 patients with histologically confirmed endometrial cancer. OS was defined as the time from diagnosis to death or last follow-up. During a median follow-up of 51.5 months (IQR, 16.0–112.8), 51 deaths were observed. Median OS was 103 months (95% CI, 37.6–168.4), and the 1-, 3-, and 5-year overall survival rates were 88%, 71%, and 54%, respectively. The shaded band represents the 95% CI, tick marks indicate censored observations, and the numbers below the plot indicate patients at risk at each time point. **Abbreviations:** CI, confidence interval; IQR, interquartile range; OS, Overall survival.

### Statistical analysis

All statistical analyses were performed using IBM SPSS Statistics version 27.0 (IBM Corp., Armonk, NY, USA). Continuous variables are presented as mean ± standard deviation or median (interquartile range), as appropriate, and categorical variables as frequencies and percentages. Group comparisons were conducted using the chi-square test, Student’s *t*-test, or the Mann–Whitney *U* test, as appropriate. Survival was estimated using the Kaplan–Meier method, and hazard ratios (HRs) with 95% confidence intervals (CIs) were calculated using Cox proportional hazards models. SINI, SII, and CA-125 values were rescaled per 100-unit increments prior to Cox regression analyses. Variables included in the multivariable Cox regression analyses were selected based on clinical relevance, univariate findings, and the limited number of events to minimize model overfitting and unstable estimates. Kaplan–Meier figures, number-at-risk tables, and a bootstrap calibration analysis for 3-year overall survival using 200 bootstrap resamples were generated using R software (R Foundation for Statistical Computing, Vienna, Austria). A standard receiver operating characteristic (ROC) curve analysis based on survival status was performed to determine the optimal cut-off value for SINI using Youden’s index. A two-sided *P*-value <0.05 was considered statistically significant.

## Results

A total of 125 patients with histologically confirmed endometrial cancer treated at Manisa Celal Bayar University between March 2011 and October 2024 were assessed for eligibility. After excluding patients due to missing laboratory data (*n* ═ 13) or inadequate follow-up/follow-up outside our institution (*n* ═ 8), 104 consecutive eligible patients were included in the final analysis. The mean age was 61.6 ± 10.6 years, and the mean body mass index was 31.9 ± 6.6 kg/m^2^. Most patients presented with vaginal bleeding (79.8%) and underwent primary surgery (86.5%), with lymph node dissection performed in 72.1%. At diagnosis, 17.3% of patients had stage I disease, 12.5% stage II, 37.5% stage III, and 32.7% stage IV disease. Endometrioid histology accounted for 59.6% of cases, and lymphovascular space invasion was present in 35.3%.

Exploratory ROC curve analysis demonstrated that SINI significantly discriminated overall survival (area under the curve [AUC]: 0.633; 95% CI: 0.525–0.741; *P ═* 0.020). A potential cut-off value of 159.3 was identified for patient stratification using the maximum Youden index (0.254), corresponding to a sensitivity of 74.5% and specificity of 50.9%. Patients were stratified into low and high SINI groups, comprising 27 (26.0%) and 77 (74.0%) patients, respectively. Patients with SINI >159.3 were significantly older (mean age 62.9 vs. 57.5 years; *P ═* 0.022) and more frequently presented with advanced-stage disease (*P ═* 0.024), non-endometrioid histology (*P ═* 0.025), and higher-grade tumors (*P ═* 0.039). Detailed clinicopathological characteristics according to SINI groups are presented in Table 1.

### Survival outcomes

The median follow-up duration for the cohort was 51.5 months (interquartile range [IQR]: 16–112.8 months), during which 51 death events were recorded. The estimated median OS was 103 months (95% CI: 37.6–168.4 months), as depicted in Figure 1. The 1-, 3-, and 5-year OS rates for the entire cohort were 88%, 71%, and 54%, respectively.

For exploratory survival analyses, patients were stratified into two groups based on a SINI cut-off value of 159.3: a low SINI group (SINI ≤159.3) and a high SINI group (SINI >159.3). Of the observed death events, 8 (15.7%) occurred in the low SINI group, while 43 (84.3%) occurred in the high SINI group. Patients in the low SINI group exhibited a significantly longer mean OS (134.9 months; 95% CI: 111.4–158.5 months) compared to those in the high SINI group (97.1 months; 95% CI: 75.2–119.0 months) (Figure 2). Furthermore, the 1-, 3-, and 5-year OS rates were notably higher in the low SINI group (96%, 80%, and 80%, respectively) than in the high SINI group (85%, 51%, and 42%, respectively), with this difference reaching statistical significance (log-rank *P ═* 0.006).

**Figure 2.**
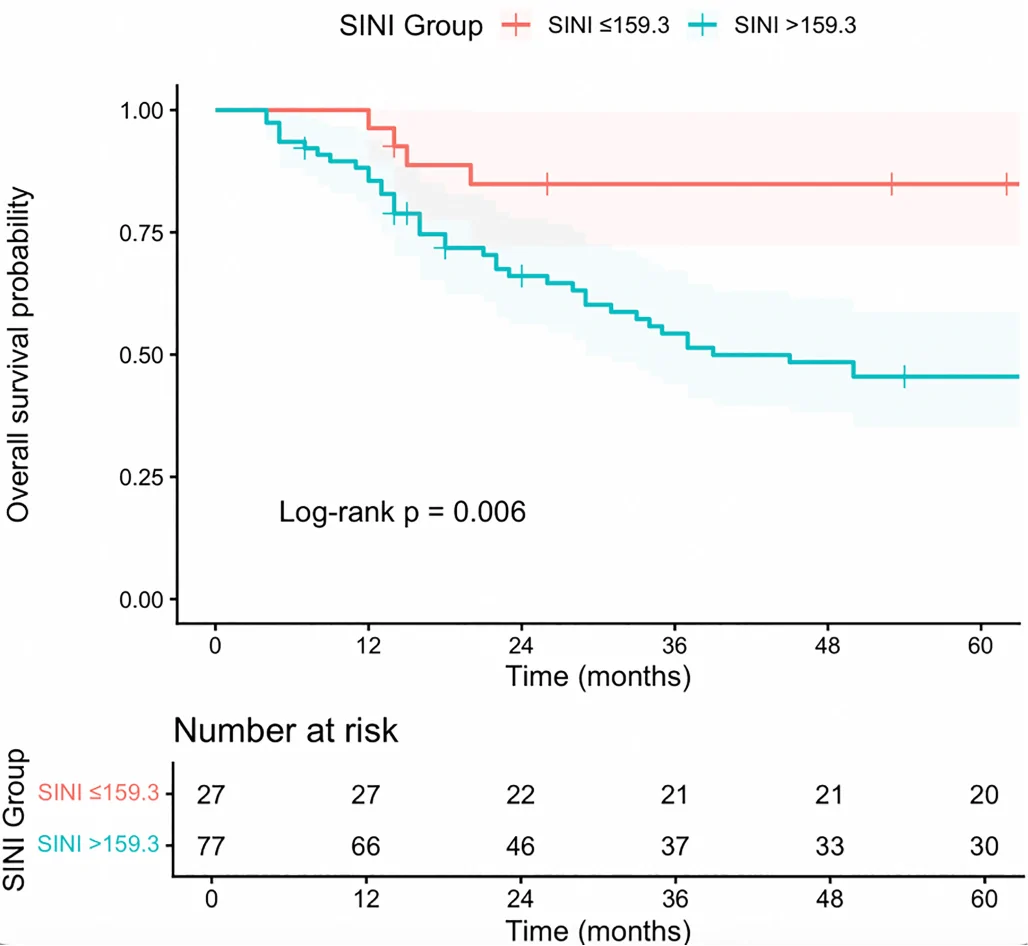
**High SINI identifies patients with shorter overall survival.** Kaplan–Meier estimates of overall survival according to the exploratory ROC-derived SINI cutoff in patients with endometrial cancer (*n* ═ 104). SINI was calculated as platelet count × neutrophil count/lymphocyte count × serum albumin level, divided by 100 for analysis, and dichotomized as low SINI (≤159.3; *n* ═ 27) or high SINI (>159.3; *n* ═ 77). Overall survival was defined as the time from diagnosis to death or last follow-up. Median follow-up was 51.5 months (IQR, 16.0–112.8); 8 deaths occurred in the low SINI group and 43 deaths in the high SINI group. Estimated mean overall survival was longer in the low SINI group than in the high SINI group (134.9 months [95% CI, 111.4–158.5] vs. 97.1 months [95% CI, 75.2–119.0]). The 1-, 3-, and 5-year overall survival rates were 96%, 80%, and 80% in the low SINI group and 85%, 51%, and 42% in the high SINI group, respectively (log-rank test, *P ═* 0.006). Shaded bands represent 95% CIs, tick marks indicate censored observations, and numbers below the plot indicate patients at risk at each time point. **Abbreviations:** CI, confidence interval; IQR, interquartile range; ROC, receiver operating characteristic; SINI, systemic inflammation and nutritional index.

PFS analysis revealed 46 progression events during follow-up, with 7 events in the SINI ≤159.3 group and 39 events in the SINI >159.3 group. The 1-, 3-, and 5-year PFS rates were 85%, 73%, and 73% in the low SINI group, vs 77%, 51%, and 44% in the high SINI group (log-rank *P ═* 0.018) (Figure 3).

**Figure 3.**
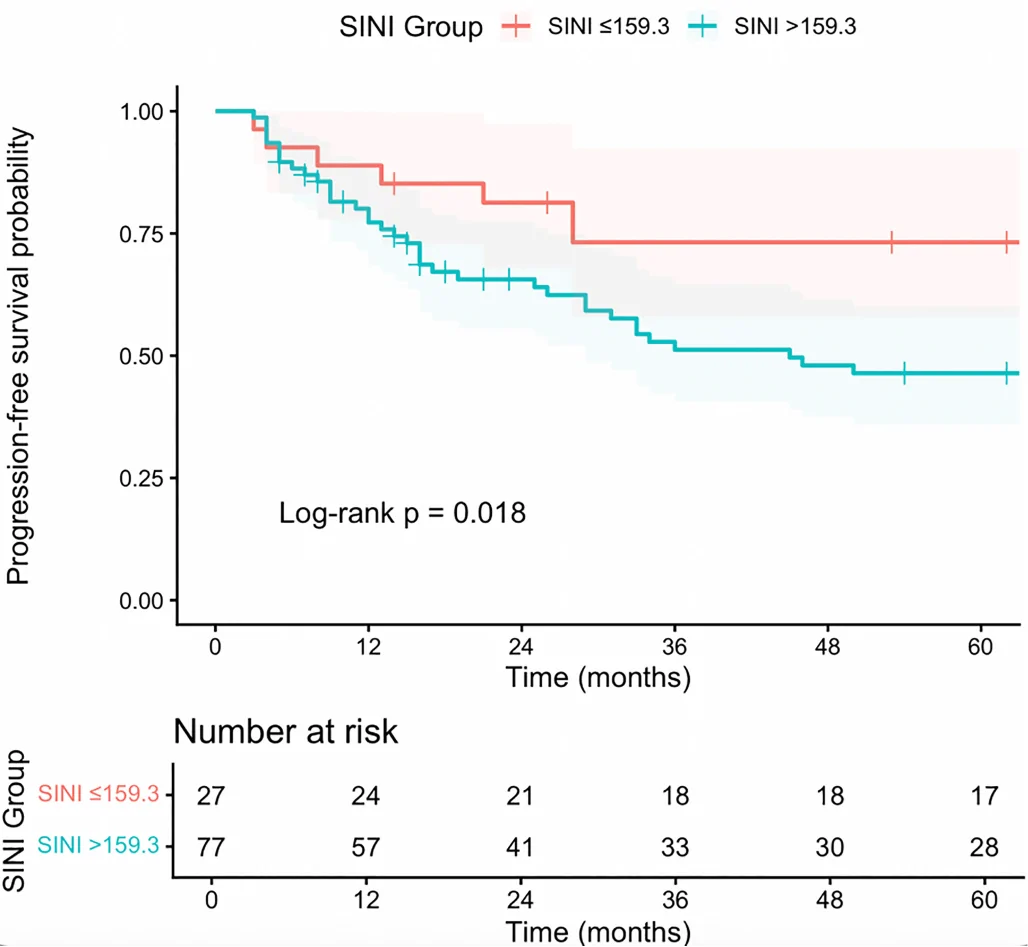
**High SINI is associated with shorter PFS.** Kaplan–Meier estimates of PFS according to the exploratory ROC-derived SINI cutoff in patients with endometrial cancer (*n* ═ 104). SINI was calculated as platelet count × neutrophil count/lymphocyte count × serum albumin level, divided by 100 for analysis, and patients were stratified into low SINI (≤159.3; *n* ═ 27) and high SINI (>159.3; *n* ═ 77) groups. PFS was defined as the time from diagnosis to disease progression, recurrence, or death from any cause. During a median follow-up of 51.5 months (IQR, 16.0–112.8), 46 progression events were observed, including 7 events in the low SINI group and 39 events in the high SINI group. The 1-, 3-, and 5-year PFS were 85%, 73%, and 73% in the low SINI group and 77%, 51%, and 44% in the high SINI group, respectively (log-rank test, *P ═* 0.018). Shaded bands represent 95% CIs, tick marks indicate censored observations, and numbers below the plot indicate patients at risk at each time point. **Abbreviations:** CI, confidence interval; IQR, interquartile range; PFS, progression-free survival; ROC, receiver operating characteristic; SINI, systemic inflammation and nutritional index.

### Univariate and multivariate analyses of overall survival

Univariate Cox regression analysis identified several clinicopathological factors significantly associated with worse OS: positive lymph nodes (HR: 1.10; 95% CI: 1.042–1.156; *P <* 0.001), non-endometrioid histology (HR: 2.27; 95% CI: 1.302–3.954; *P ═* 0.004), Grade 2 disease (HR: 6.15; 95% CI: 1.376–27.485; *P ═* 0.017), Grade 3 disease (HR: 7.29; 95% CI: 1.659–32.022; *P ═* 0.009), and the presence of LVSI (HR: 5.86; 95% CI: 1.715–20.030; *P ═* 0.005). Conversely, primary surgery (HR: 0.29; 95% CI: 0.150–0.577; *P <* 0.001), adjuvant radiotherapy (HR: 0.37; 95% CI: 0.208–0.640; *P <* 0.001), and adjuvant chemotherapy (HR: 0.36; 95% CI: 0.199–0.635; *P <* 0.001) were associated with improved OS.

SINI was significantly associated with OS (HR: 1.065; 95% CI: 1.019–1.112; *P ═* 0.005). Specifically, patients with SINI >159.3 demonstrated a significantly increased mortality risk in univariate Cox regression analysis (HR: 2.75; 95% CI: 1.287–5.886; *P ═* 0.009). Among laboratory parameters, higher SII (HR: 1.015; 95% CI: 1.003–1.026; *P ═* 0.011), higher Ki-67 index (HR: 1.01; 95% CI: 1.001–1.024; *P ═* 0.030), elevated CA-125 levels (HR: 1.044; 95% CI: 1.011–1.078; *P ═* 0.012), and lower albumin levels (HR: 0.54; 95% CI: 0.341–0.863; *P ═* 0.010) were also significantly associated with worse OS (Table 2).

**Table 2:** Univariate and multivariate analyses of overall survival

	**Univariate**			**Multivariate**		
	**HR**	**95% CI**	***P*-value**	**HR**	**95% CI**	***P*-value**
Age	1.020	0.993–1.049	0.152			
ECOG	0.582	0.283–1.196	0.141			
BMI (kg/m^2^)	1.010	0.967–1.056	0.644			
Operation (Ref: No)	0.294	0.150–0.577	<0.001			
Adjuvant Radiotherapy	0.365	0.208–0.640	<0.001			
Adjuvant Chemotherapy	0.356	0.199–0.635	<0.001		
Lymph Node Dissection (Ref: No)	0.607	0.338–1.088	0.094			
Positive Lymph Nodes	1.098	1.042–1.156	<0.001			
Ki-67 index (%)	1.013	1.001–1.024	0.030			
Histopathology (Ref: endometrioid)	2.269	1.302–3.954	0.004	2.203	1.233–3.935	0.008
Grade (Ref: Grade 1)			0.027			
Grade 2	6.149	1.376–27.485	0.017			
Grade 3	7.288	1.659–32.022	0.009			
LVSI (Ref: No)			<0.001			
Yes	5.860	1.715–20.030	0.005			
Unknown	13.083	3.893–43.970	<0.001			
Myometrial invasion (Ref: <50%)			0.687			
>50%	1.233	0.663–2.293	0.508			
Unknown	0.777	0.176–3.426	0.739			
CA-125 (per 100 units)	1.044	1.011–1.078	0.012	1.025	0.990–1.061	0.171
CEA	0.973	0.891–1.063	0.542			
NLR	1.035	0.993–1.078	0.101			
Platelet	1.001	0.999–1.003	0.332			
Albumin	0.543	0.341–0.863	0.010			
NLR x albumin	1.008	0.997–1.019	0.140			
PLR x albumin	1.016	0.993–1.040	0.181			
SII (per 100 units)	1.015	1.003–1.026	0.011			
SINI (per 100 units)	1.065	1.019–1.112	0.005			
SINI> 159.3	2.752	1.287–5.886	0.009	2.893	1.302–6.429	0.009
*De novo* metastasis	9.178	4.875–17.279	<0.001			
Liver metastasis	3.908	2.157–7.080	<0.001	2.733	1.407–5.308	0.003
Lung metastasis	3.650	2.079–6.408	<0.001	3.081	1.605–5.914	< 0.001
Bone metastasis	3.180	1.410–7.170	0.005			
Brain metastasis	2.298	0.555–9.510	0.251			

**Abbreviations:** BMI, body mass index; CA-125, cancer antigen 125; CEA, carcinoembryonic antigen; CI, confidence interval; ECOG, Eastern Cooperative Oncology Group performance status; HR, hazard ratio; LVSI, lymphovascular space invasion; NLR, neutrophil-to-lymphocyte ratio; PLR, platelet-to-lymphocyte ratio; Ref, reference; SII, systemic-immune inflammation index; SINI, systemic inflammation and nutritional index.

In an exploratory cut-off based multivariate Cox regression analysis, SINI >159.3 remained an independent predictor of shorter OS (HR: 2.89; 95% CI: 1.302–6.429; *P ═* 0.009). Other significant risk factors for worse OS included liver metastasis (HR: 2.73; 95% CI: 1.407–5.308; *P ═* 0.003), lung metastasis (HR: 3.08; 95% CI: 1.605–5.914; *P <* 0.001), and histopathology (HR: 2.20; 95% CI: 1.233–3.935; *P ═* 0.008). Notably, CA-125 lost its statistical significance after adjustment in this multivariate model (Table 2).

To further elucidate the association between SINI and overall survival, a sensitivity analysis was conducted utilizing a simplified disease-stage grouping (Stage IV vs Stages I–III at diagnosis) alongside major treatment variables. This analysis confirmed that higher SINI was independently associated with poorer OS (HR: 2.49, 95% CI: 1.13–5.47, *P ═* 0.023). Stage IV disease at diagnosis also predicted worse OS (HR: 2.35, 95% CI: 1.15–4.81, *P ═* 0.019), while adjuvant chemotherapy was associated with improved survival (HR: 0.45, 95% CI: 0.23–0.90, *P ═* 0.023). Conversely, surgery (HR: 0.82, 95% CI: 0.37–1.83, *P ═* 0.627) and adjuvant radiotherapy (HR: 0.73, 95% CI: 0.36–1.48, *P ═* 0.388) did not demonstrate a significant association with OS in this sensitivity analysis.

### Univariate and multivariate analyses of progression-free survival

Univariate Cox regression analysis for PFS identified several clinicopathological factors associated with disease progression: an increasing number of positive lymph nodes (HR: 1.06; 95% CI: 1.010–1.114; *P ═* 0.017), de novo metastasis (HR: 32.77; 95% CI: 12.377–86.743; *P ═* 0.001), and the presence of liver (HR: 4.56; 95% CI: 2.482–8.374; *P <* 0.001), lung (HR: 4.35; 95% CI: 2.460–7.696; *P <* 0.001), and bone metastases (HR: 4.00; 95% CI: 1.842–8.679; *P <* 0.001). In contrast, primary surgery (HR: 0.30; 95% CI: 0.154–0.578; *P <* 0.001) and lymph node dissection (HR: 0.56; 95% CI: 0.317–0.980; *P ═* 0.042) were associated with improved PFS. Examination of laboratory variables revealed that SINI >159.3 was a significant risk factor for disease progression (HR: 2.54; 95% CI: 1.132–5.714; *P ═* 0.024). Elevated CA-125 levels were also associated with an increased risk of progression (HR: 1.03; 95% CI: 1.003–1.066; *P ═* 0.031), while other laboratory markers did not show a significant relationship (Table 3).

**Table 3:** Univariate and multivariate analyses of progression-free survival

	**Univariate**			**Multivariate**		
	**HR**	**95% CI**	***P*-value**	**HR**	**95% CI**	***P*-value**
Age	1.013	0.986–1.040	0.358			
ECOG	0.566	0.284–1.129	0.106			
BMI (kg/m^2^)	1.011	0.970–1.054	0.617			
Operation (Ref: No)	0.298	0.154–0.578	<0.001			
Adjuvant Radiotherapy	0.270	0.147–0.494	<0.001			
Adjuvant Chemotherapy	0.271	0.144–0.510	<0.001			
Lymph Node Dissection (Ref: No)	0.558	0.317–0.980	0.042			
Positive Lymph Nodes	1.061	1.010–1.114	0.017			
Ki67 index (%)	1.008	0.997–1.019	0.171			
Histopathology (Ref: endometrioid)	1.534	0.890–2.642	0.123			
Grade (Ref: Grade 1)			0.334			
Grade 2	2.617	0.851–8.052	0.093			
Grade 3	2.558	0.839–7.800	0.099			
LVSI (Ref: No)			0.001			
Yes	2.139	0.829–5.519	0.116			
Unknown	4.621	1.911–11.175	<0.001			
Myometrial invasion (Ref: <50%)			0.687			
>50%	1.095	0.597–2.012	0.769			
Unknown	0.603	0.138–2.638	0.502			
CA-125 (per 100 units)	1.034	1.003–1.066	0.031	1.017	0.983-1.052	0.340
CEA	1.033	0.978–1.090	0.241			
NLR	1.017	0.972–1.064	0.460			
Platelet	1.001	0.999–1.004	0.398			
Albumin	0.692	0.422–1.133	0.143			
NLR x albumin	1.005	0.994–1.017	0.369			
PLR x albumin	1.011	0.984–1.038	0.430			
SII (per 100 units)	1.010	0.997–1.023	0.136			
SINI (per 100 units)	1.042	0.991–1.096	0.110			
SINI >159.3	2.543	1.132–5.714	0.024	2.490	1.101–5.628	0.028
*De novo* metastasis	32.766	12.377–86.743	0.001			
Liver metastasis	4.559	2.482–8.374	<0.001	2.368	1.167–4.805	0.017
Lung metastasis	4.351	2.460–7.696	<0.001	3.919	2.040–7.527	<0.001
Bone metastasis	3.998	1.842–8.679	<0.001			
Brain Metastasis	2.305	0.556–9.546	0.250			

**Abbreviations:** BMI, body mass index; CA-125, cancer antigen 125; CEA, carcinoembryonic antigen; CI, confidence interval; ECOG, Eastern Cooperative Oncology Group performance status; HR, hazard ratio; LVSI, lymphovascular space invasion; NLR, neutrophil-to-lymphocyte ratio; PLR, platelet-to-lymphocyte ratio; Ref, reference; SII, systemic-immune inflammation index; SINI, systemic inflammation and nutritional index.

In an exploratory cut-off based multivariate Cox regression analysis, SINI >159.3 remained independently associated with disease progression (HR: 2.490; 95% CI: 1.101–5.628; *P ═* 0.028). Additionally, the presence of liver metastasis (HR: 2.368; 95% CI: 1.167–4.805; *P ═* 0.017) and lung metastasis (HR: 3.919; 95% CI: 2.040–7.527; *P <* 0.001) continued to be significantly associated with shorter PFS. Other variables, including CA-125, did not retain statistical significance after adjustment in this model (Table 3).

### Comparative model performance of SINI, SII, and NLR

In multivariable Cox regression analysis using continuous variables, both SINI (*P ═* 0.002) and SII (*P ═* 0.017) demonstrated significant associations with survival outcomes, whereas the NLR did not achieve statistical significance (*P ═* 0.473). Regarding model performance, SINI exhibited the lowest −2 log likelihood and Akaike Information Criterion (AIC) values (380.075 and 390.075, respectively), indicating a superior model fit compared to SII (385.950 and 395.950) and NLR (389.775 and 399.775). Consistently, SINI also yielded the highest C-index (0.652), outperforming SII (0.640) and NLR (0.633), suggesting modest differences in discriminative ability. Bootstrap calibration analysis confirmed generally acceptable agreement between predicted and observed 3-year OS probabilities for SINI, although some deviation was noted in the lower risk range (Figure S1).

## Discussion

Endometrial cancer is a clinically heterogeneous disease, necessitating the identification of additional biomarkers beyond conventional models for accurate prognostic assessment. Li et al. conducted a large retrospective study of 1,483 patients, demonstrating that a preoperative CA125 level ≥35 U/mL independently correlated with diminished progression-free and overall survival. This finding led to its integration into a prognostic nomogram alongside inflammatory and nutritional markers. Furthermore, the study highlighted CA125’s routine application in monitoring disease progression and treatment response, underscoring its clinical utility as a biomarker [12].

Among various inflammation-based indices, the SII has garnered significant attention across multiple endometrial cancer cohorts. Matsubara et al.’s study of 442 patients revealed that elevated pre-treatment SII independently correlated with shorter progression-free and overall survival. Their analysis, based on ROC curves, indicated that SII exhibited superior predictive performance compared to the NLR and platelet-to-lymphocyte ratio (PLR) [13]. A subsequent meta-analysis corroborated these findings, reporting a significant association between elevated SII and poorer overall and progression-free survival in endometrial cancer patients [14]. Additionally, an SII ≥494.26 has been identified as an independent predictor of suboptimal response to neoadjuvant chemotherapy in endometrial cancer and has been incorporated into prognostic models for survival estimation [15, 16].

Concurrently, serum albumin has been specifically investigated as a prognostic biomarker in endometrial cancer. A cohort study of 337 patients revealed that pre-treatment serum albumin levels were independently associated with both disease-free and progression-free survival, exhibiting an inverse correlation with FIGO stage and tumor grade [17]. These findings are further supported by studies involving larger populations and composite indices integrating nutritional and inflammatory parameters. A meta-analysis encompassing 9,428 patients with gynecological cancers demonstrated that a low prognostic nutritional index (PNI) was associated with adverse survival outcomes [18]. Similarly, a high C-reactive protein (CRP)/albumin ratio correlated with poorer survival, disease progression, and advanced-stage disease [19]. Collectively, these data underscore albumin’s role as a marker reflecting both nutritional status and systemic inflammation.

In the current study, we developed the SINI, which integrates platelet count, neutrophil-to-lymphocyte ratio, and serum albumin levels. Each of these components has previously demonstrated an association with survival outcomes in endometrial cancer. Our cohort analysis indicated that higher SINI values were associated with poorer survival, suggesting that the synergistic combination of inflammatory and nutritional parameters into a single index may offer enhanced prognostic utility in this clinical context.

### Limitations

This study acknowledges several limitations. First, its retrospective design inherently introduces potential selection bias and unmeasured confounding factors, such as active infection, chronic inflammatory disease, liver disease, hematologic disorders, or steroid use. Furthermore, molecular and immunohistochemical data were not consistently available throughout the study period due to the retrospective nature of the design. Second, the relatively limited sample size may have reduced statistical power, potentially restricting the generalizability of the findings, particularly to early-stage endometrial cancer populations. The limited number of events may also have constrained the inclusion of all clinically relevant variables within a single multivariable model. Moreover, the SINI cut-off value was determined using standard ROC analysis and subsequently evaluated within the same cohort, which carries a risk of overfitting and may limit its applicability to external populations. While SINI demonstrated relatively improved model performance compared to SII and NLR, the ROC and C-index analyses indicated only modest discriminative ability. Consequently, the cut-off-based analyses should be interpreted as exploratory, and external validation in larger independent cohorts is warranted. A basic bootstrap-based internal calibration analysis demonstrated generally acceptable agreement between predicted and observed survival probabilities; however, these findings should be interpreted cautiously due to the limited sample size and absence of external validation. Prospective multicenter studies are imperative to confirm these findings.

Despite these limitations, this study possesses notable strengths. We developed a novel composite index, the SINI, which integrates platelet count, neutrophil-to-lymphocyte ratio, and serum albumin levels. This index leverages inexpensive and routinely available parameters, enhancing its practicality and accessibility in diverse clinical settings. While individual markers such as NLR, SII, and serum albumin have been previously investigated as prognostic factors in endometrial cancer, these parameters reflect only isolated aspects of host physiology. SINI, conversely, integrates both inflammatory burden and nutritional status into a single composite score, potentially offering a more comprehensive reflection of the systemic host response. In comparative model performance analyses, SINI demonstrated improved model fit compared to SII and NLR, evidenced by lower AIC values and the highest C-index. Although the differences in AIC and C-index values between SINI and existing inflammatory/nutritional markers were modest, SINI may still provide a more integrated assessment of systemic inflammation and nutritional status by combining routinely available parameters within a single composite index.

## Conclusion

In conclusion, SINI was associated with overall survival in this retrospective cohort of patients with endometrial cancer. As a composite index integrating inflammatory and nutritional parameters derived from routinely available laboratory variables, SINI may offer prognostic utility in this setting. However, given the retrospective single-center design, modest discriminative performance, and absence of external validation, these findings necessitate cautious interpretation. Prospective multicenter studies are required to confirm these results before SINI can be recommended for routine clinical use. Further research is also needed to investigate whether immune-nutritional indices may have utility in guiding therapeutic strategies.

## Supplemental data


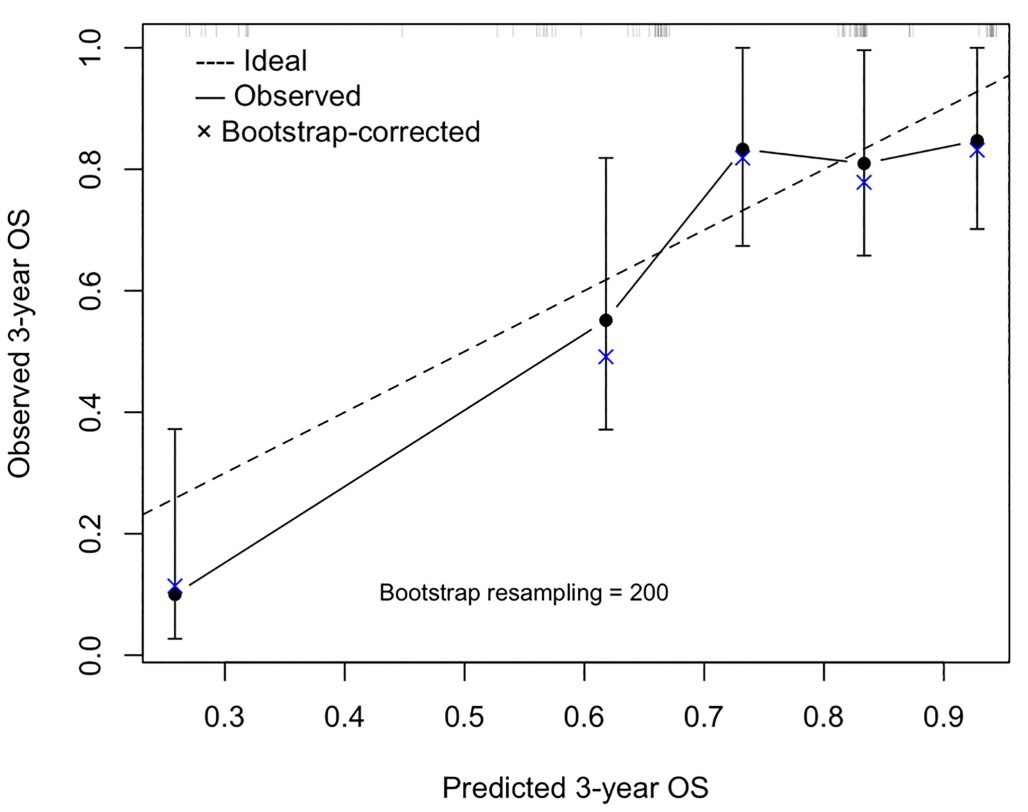


**Figure S1. Bootstrap calibration plot for 3-year overall survival.** Calibration of the SINI-based prognostic model for predicting 3-year OS. The dashed diagonal line represents ideal agreement between predicted and observed survival probabilities, while the solid line shows the observed calibration curve. Blue crosses indicate bootstrap-corrected estimates obtained using 200 bootstrap resamples, and vertical bars reflect uncertainty around the observed estimates. The model demonstrated generally acceptable calibration across most predicted probabilities, with some deviation in the lower predicted-probability range. **Abbreviations:** OS, overall survival; SINI, systemic inflammation and nutritional index.

## Data Availability

The datasets generated and analyzed during the current study are not publicly available but are available from the corresponding author upon reasonable request.
